# Immune hyperactivity in hemodialysis patients is associated with interferon gamma-induced trained immunity

**DOI:** 10.1016/j.isci.2026.116496

**Published:** 2026-06-23

**Authors:** Inge Jonkman, Maaike M.E. Jacobs, Leonie S. Helder, Yutaka Negishi, Jordi Ochando, Joren C. Madsen, Musa M. Mhlanga, Abraham J.P. Teunissen, Leo A.B. Joosten, Mihai G. Netea, Luuk B. Hilbrands, Nils Rother, Raphaël Duivenvoorden

**Affiliations:** 1 Department of Nephrology, Radboud University Medical Center, Nijmegen, the Netherlands; 2 Department of Internal Medicine and Radboud Community for Infectious Diseases, Radboud University Medical Center, Nijmegen, the Netherlands; 3 Department of Cell Biology, Faculty of Science, Radboud University, Nijmegen, the Netherlands; 4 Department of Human Genetics, Radboud University Medical Center, Nijmegen, the Netherlands; 5 Department of Oncological Sciences, Icahn School of Medicine at Mount Sinai, New York, NY, USA; 6 Transplant Immunology Unit, National Center of Microbiology, Instituto de Salud Carlos III, Madrid, Spain; 7 Center for Transplantation Sciences, Department of Surgery, Massachusetts General Hospital, Boston, MA, USA; 8 Division of Cardiac Surgery, Department of Surgery, Massachusetts General Hospital, Boston, MA, USA; 9 BioMedical Engineering and Imaging Institute, Icahn School of Medicine at Mount Sinai, New York, NY, USA; 10 Cardiovascular Research Institute, Icahn School of Medicine at Mount Sinai, New York, NY, USA; 11 Icahn Genomics Institute, Icahn School of Medicine at Mount Sinai, New York, NY, USA; 12 Department of Medical Genetics, Iuliu Haţieganu University of Medicine and Pharmacy, Cluj-Napoca, Romania; 13 Department of Immunology and Metabolism, Life and Medical Sciences Institute, University of Bonn, Bonn, Germany

**Keywords:** immunology, nephrology, molecular medicine, cell biology, systems biology

## Abstract

Hemodialysis patients experience persistent inflammation marked by pro-inflammatory monocytes. We hypothesized that the hyper-responsiveness of innate immune cells in these patients is facilitated by trained immunity, a form of innate immune memory. Hemodialysis patients displayed elevated monocyte counts, and isolated peripheral blood mononuclear cells showed significantly heightened cytokine responses after Toll-like receptor stimulation, both indicative of trained immunity. Importantly, plasma interferon gamma (IFN-γ) concentrations positively correlated with cytokine responses. Whole-genome RNA-sequencing revealed enrichment of interferon response pathways, particularly in patients whose monocytes exhibited the most pronounced cytokine production upon restimulation. *In vitro* experiments confirmed that trained immunity induction depends on IFN-γ, produced by CD4^+^ T cells. Our findings demonstrate that hemodialysis patients display a dysregulated immune response characterized by trained immunity and that this might be mediated by IFN-γ. These insights suggest that targeting IFN-γ could be a promising strategy to mitigate damaging immune hyperactivity in dialysis patients.

## Highlights

• Hemodialysis patient PBMCs show enhanced cytokine responses consistent with trained immunity
• Plasma Interferon gamma levels in hemodialysis patients correlate with cytokine responses
• Transcriptomic analysis revealed increased expression of interferon pathway genes
• *In vitro* trained immunity is enhanced by interferon gamma produced by proliferating T cells

## Graphical abstract

**Figure:**
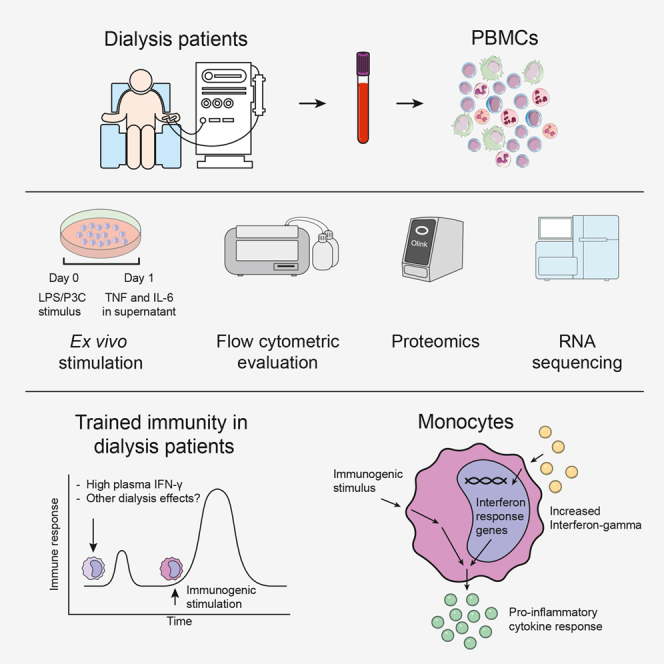

## Introduction

Patients with chronic kidney disease (CKD) and those undergoing dialysis have significantly higher mortality rates and face an increased risk of infections and immune-mediated diseases, including virus-associated cancers, periodontitis, and atherosclerosis, compared to the general population.^1^^,^^2^^,^^3^^,^^4^ A key factor contributing to these poor outcomes is chronic low-grade inflammation facilitated by immune dysregulation.^5^ While the precise mechanisms behind this immune dysregulation remain unclear, CKD and dialysis are linked to an increased production of pro-inflammatory monocytes in the bone marrow.^6^^,^^7^^,^^8^ This heightened inflammatory profile is accompanied by elevated concentrations of circulating cytokines, including interleukin (IL)-6, IL-1β, and tumor necrosis factor (TNF).^9^^,^^10^^,^^11^^,^^12^ Remarkably, this pro-inflammatory activity persists even after uremia is resolved following kidney transplantation.^13^

We hypothesize that this enhanced pro-inflammatory profile of innate immune cells in CKD and dialysis patients is facilitated by innate immune memory, also known as trained immunity.^14^ Trained immunity refers to the phenomenon in which innate immune cells develop an increased responsiveness following an initial stimulus, resulting in an enhanced cytokine response to secondary stimulation.^15^ Trained immunity can be induced in circulating myeloid and lymphoid innate immune cells as well as in their bone marrow progenitors, leading to the durable production of trained cells.^16^ Although trained immunity has been studied in various diseases, including infections, auto-inflammatory disorders, gout, cancer, atherosclerosis, and kidney transplantation, its role in driving the persistent inflammation associated with CKD and dialysis remains unexplored.^14^^,^^17^

In this study, we sought to determine whether trained immunity manifests in hemodialysis patients and, if so, to unravel the molecular mechanisms associated with its induction.

## Results

### PBMCs of hemodialysis patients display hyperresponsiveness

**Figure 1:**
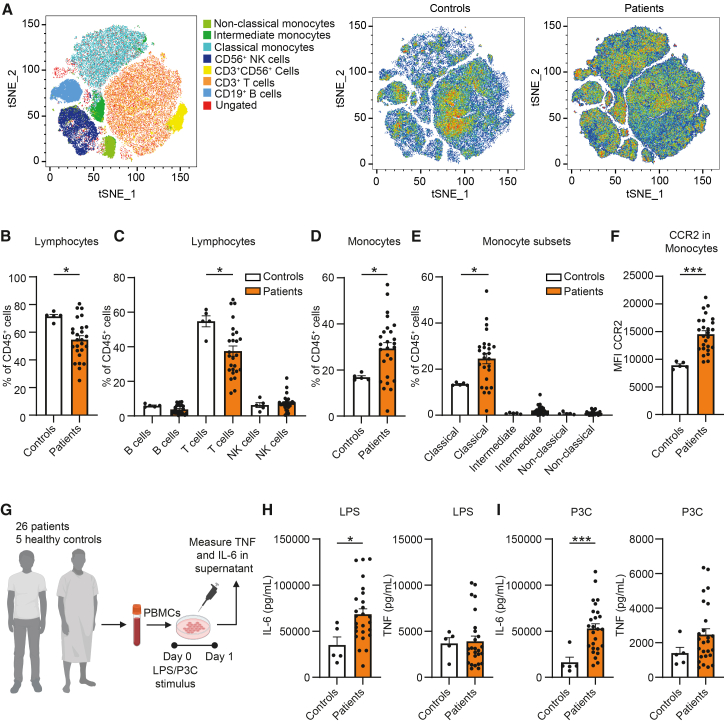
Immune cell profile and response of PBMCs obtained from hemodialysis (HD) patients versus healthy controls (A) t-distributed stochastic neighbor embedding (t-SNE) plots show unsupervised clustering on the expression of 10 markers (CD45, CD14, CD16, CD3, CD19, CD56, HLA-DR, CD11b, CCR2, and CX3CR1) in PBMCs of HD patients (*n* = 26) and healthy controls (*n* = 5) measured by flow cytometry. (B and C) Quantification of lymphocytes and lymphocyte subsets in PBMCs of HD patients (*n* = 26) and healthy controls (*n* = 5). (D and E) Quantification of monocytes and monocyte subsets (classical CD14^+^CD16^−^, intermediate CD14dimCD16+, and non-classical CD14^+^CD16^+^ monocytes) in PBMCs of HD patients (*n* = 26) and healthy controls (*n* = 5). (F) Expression of monocyte activation marker CCR2 in monocytes of HD patients (*n* = 26) and healthy controls (*n* = 5). (G) Schematic representation of material collection and subsequent *in vitro* assay. (H and I) Interleukin 6 (IL-6) and tumor necrosis factor (TNF) production by PBMCs from HD patients (*n* = 26) and healthy controls (*n* = 5) incubated for 24 h with lipopolysaccharide (LPS) (H) or Pam3CSK4 (P3C) (I). Mean ± SEM. (*n* = 5 healthy controls, *n* = 26 HD patients) ∗*p* < 0.05, and ∗∗∗*p* < 0.001. Two-tailed t tests (B–F). Two-tailed t tests on log10-transformed cytokine concentrations (H, I). MFI: median fluorescence intensity. See also Figures S1–S3.

Patient characteristics are described in Table 1. The mean age was 67.5 years (SD 14.7), 61% were male. We investigated the immune cell subsets of circulating leukocytes in hemodialysis patients and compared them to those in peripheral blood mononuclear cells (PBMCs) of healthy controls (Figure 1A; Table S1; Figure S1). Hemodialysis patients had a lower percentage of lymphocytes within the CD45^+^ PBMCs, mainly due to a reduced proportion of T cells. There was a trend toward fewer B cells, while the percentage of natural killer (NK) cells was similar in patients versus controls (Figures 1B and 1C). The proportion of monocytes was markedly higher in hemodialysis patients compared to healthy controls, which was primarily due to an increase in CD14^+^CD16^−^ classical monocytes (Figures 1D and 1E). Monocyte expression of C-C chemokine receptor 2 (CCR2), a marker of monocyte activation, was increased in hemodialysis patients as compared to controls (Figure 1F). Expression of human leukocyte antigen-DR isotype (HLA-DR) and CX3C motif chemokine receptor 1 (CX3CR1) on monocytes was not different, while the expression of CD11b was decreased (Figure S2).

**Table 1:** Demographics and characteristics of dialysis patients

Characteristics	*N* = 26
Age, years	67.5 (59.3–75.5)
Male, no. (%)	16 (61.5%)
BMI	24.7 (21.9–27.0)
Systolic blood pressure (mmHg)	143 (27.1)
Diastolic blood pressure (mmHg)	69 (15.1)
Hospital admissions last year	1 (0–2)
Charlson Comorbidity Index	6 (1.5)

**History**	

Cardiovascular disease, no. (%)	19 (73.1%)
Diabetes, no. (%)	9 (34.6%)
Glomerulonephritis or autoimmune disease, no. (%)	2 (7.7%)
Cancer, no. (%)	3 (11.5%)
Previous kidney transplant, no. (%)	6 (23.1%)

**Medication use**	

Immunosuppressant use, no. (%)	9 (34.6%)
Corticosteroids, no. (%)	9 (34.6%)
ACEi/ARB, no. (%)	7 (26.9%)
Statin, no. (%)	10 (38.5%)
Glucose lowering drugs, no. (%)	8 (30.8%)
Insulin, no. (%)	8 (30.8%)
Platelet aggregation inhibitor, no. (%)	12 (46.2%)
Vitamin K antagonist or DOAC, no. (%)	2 (7.7%)

**Dialysis related parameters**	

Duration on dialysis (months)	39.0 (17.5–81.5)
Frequency of dialysis (times a week)	3 (3–3)
Kt/V	1.44 (0.32)
Hemodialysis vascular access, no. (%)	
Arteriovenous graft	5 (19.2%)
Arteriovenous fistula	16 (61.5%)
Catheter	5 (19.2%)
Estimated residual diuresis	267.0 (0.0–838.8)

**Lab values**	

Albumin (mg/mmol)	33.4 (4.7)
Ureum (mg/dL)	21.8 (20.3–26.6)
C-reactive protein (mg/L)	4.0 (2.0–7.0)
PTH (pg/mL)	16.0 (7.9–24.3)
iCa (mmol/L)	1.16 (1.13–1.20)
Potassium (mEq/L)	4.9 (0.7)
Sodium (mmol/L)	134.9 (3.0)
pH	7.37 (0.04)
Bicarbonate (mmol/L)	23.4 (2.1)
Phosphorus (mmol/L)	1.47 (1.30–1.62)
Hb (mmol/L)	6.7 (0.6)
Leukocytes (10^9^/L)	7.1 (5.6–8.5)
Thrombocytes (10^9^/L)	229.5 (89.7)

Data are presented as the mean (SD) for normally distributed data, median (interquartile range) for non-normally distributed data, or number (no.) and percentage. ACEi: angiotensin-converting enzyme inhibitors; ARB: angiotensin II receptor blockers; BMI: body mass index; DOAC: direct oral anticoagulants; Hb; hemoglobin; iCa: ionized calcium; PTH: parathyroid hormone.

Next, we performed functional assays by stimulating PBMCs for 24 h *ex vivo* with the Toll-like receptor 4 (TLR4) agonist lipopolysaccharide (LPS), the TLR2 ligand Pam3CSK4, or RPMI culture medium as a negative control, and subsequently measured the IL-6 and TNF cytokine responses as a functional readout for trained immunity (Figure 1G).^18^ We found markedly elevated cytokine responses after TLR4 and TLR2 stimulation, particularly for IL-6, suggesting PBMCs are trained *in vivo* in dialysis patients (Figures 1H and 1I). We found no correlation between the percentage of monocytes in PBMCs and the IL-6 or TNF cytokine responses (Figure S3; LPS_IL-6: rho = 0.354, *p* value = 0.051; LPS_TNF: rho = 0.173, *p* value = 0.352; P3C_IL-6: rho = 0.339, *p* value = 0.062; P3C_TNF: rho = 0.290, *p* value = 0.114). Using multivariate linear regression analysis with backward elimination to identify clinical parameters (age, sex, Charlson Comorbidity Index, immunosuppressants use, Kt/V, albumin, pre-dialysis serum urea level, serum C-reactive protein) associated with trained immunity cytokine responses, we found a negative association between patient immunosuppressant use and IL-6 responses to LPS stimulation (LPS_IL-6: B = 801.7, *p* value = 0.037, Table S2). The association between patient immunosuppressant use and TNF response to LPS was not significant (LPS_TNF: B = −2164.7, *p* value = 0.065; Table S2). Furthermore, we found no association between clinical parameters and IL-6 and TNF responses to Pam3CSK4 stimulation (P3C_IL-6: B = −18123.5, *p* value = 0.098; P3C_TNF: no final model; Table S2).

### Trained immunity is associated with IFN-γ signaling in hemodialysis patients

**Figure 2:**
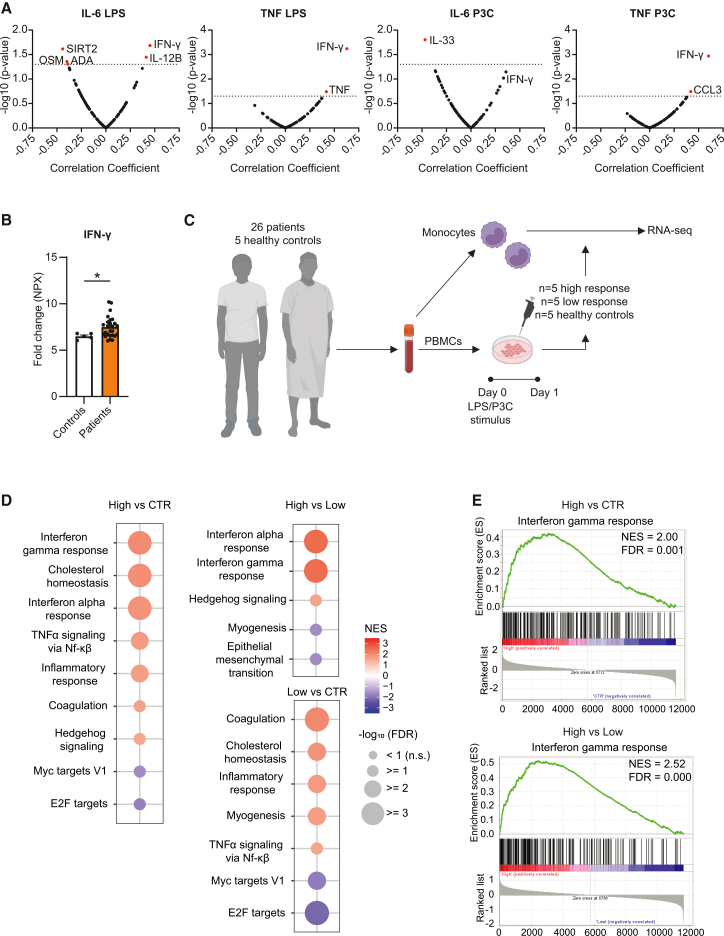
Pro-inflammatory cytokine responses of PBMCs from hemodialysis patients correlate with IFN-γ signaling (A) Volcano plot shows the correlation coefficient and *p* values of the expression of 91 inflammation-related proteins (Olink inflammation panel) measured in plasma of 26 hemodialysis patients, and interleukin 6 (IL-6) or tumor necrosis factor (TNF) production 24 h after PBMC stimulation with lipopolysaccharide (LPS) or Pam3CSK4 (P3C). *p* values were not corrected for multiple testing. (B) Interferon gamma (IFN-γ) levels measured in the plasma of hemodialysis patients and healthy controls were measured with Olink. (*n* = 5 healthy controls, *n* = 26 HD patients). (C) Schematic overview of sample collection and subsequent selection of patient samples for RNA sequencing. (D) Significantly altered gene sets of the HALLMARK database between 5 patients show high IL-6 and TNF responses to LPS or Pam3CSK4 stimulation versus 5 healthy controls (High vs. CTR), or 5 patients showing high versus low IL-6 and TNF responses to LPS or Pam3CSK4 stimulation (high vs. low), or 5 patients showing low IL-6 and TNF responses to LPS or Pam3CSK4 stimulation versus 5 healthy controls (Low vs. CTR). (E) Enrichment plot of HALLMARK gene set “interferon gamma response” in monocytes of 5 patients shows high IL-6 and TNF responses to LPS or Pam3CSK4 stimulation versus 5 healthy controls (high vs. CTR), or 5 patients showing high versus low IL-6 and TNF responses to LPS or Pam3CSK4 (High vs. Low). Mean ± SEM. (*n* = 5 healthy controls, *n* = 26 HD patients). Spearman’s rho correlation (A).∗*p* < 0.05, two-tailed *t* test (B). NES: normalized enrichment score, FDR: false discovery rate. See also Figure S4.

Next, we investigated whether the IL-6 and TNF cytokine responses measured in our cell stimulation were associated with the manifestation of inflammation in hemodialysis patients. For this purpose, the normalized protein expressions (NPXs) of 91 inflammatory proteins were measured in plasma using the Olink Proximity Extension Assay (PEA) technology (inflammation panel, Olink Bioscience, Sweden), and we correlated their expression to the IL-6 and TNF cytokine responses measured in our *in vitro* assay. Since this was an exploratory and hypothesis-generating analysis, no multiple testing correction was applied. We found that interferon gamma (IFN-γ) was the protein that showed the highest correlation with the IL-6 and TNF production of patients’ PBMCs after LPS or Pam3CSK4 stimulation (Figure 2A), making it a potential candidate to be involved in the response of patients' PBMCs. Multivariate linear regression analysis with backward elimination showed sex and Kt/V to be the most important determinants of IFN-γ concentrations (Table S3). When adjusting for clinical parameters in a multivariate linear regression analysis with backward elimination, we found that IFN-γ was an independent predictor of IL-6 and TNF cytokine production by PBMCs in response to LPS stimulation, and TNF production in response to Pam3CSK4 stimulation (Table S4). IFN-γ concentrations in plasma from hemodialysis patients were higher than in the controls (Figure 2B). TNF and IL6 cytokine responses, as well as flow cytometric data, did not correlate with the duration of dialysis or Charlson Comorbidity Index (CCI). Correlation analysis between dialysis duration as well as CCI and Olink proteomics data revealed that the duration of dialysis was correlated with the concentration of hepatocyte growth factor (HGF), and CCI was correlated with levels of urokinase (uPA), CUB-domain containing protein 1 (CDCP1), and TNF superfamily member 14 (TNFSF14) (Table S5).

Next, we isolated monocytes from PBMCs obtained from five hemodialysis patients with the lowest (low trained immunity group) and five with the highest (high trained immunity group) LPS and Pam3CSK4-induced IL-6 and TNF responses, and from the five healthy controls. Patient characteristics are described in Table S6. We analyzed the monocytes’ transcriptomes by whole-genome ribonucleic acid-sequencing (RNA-seq) and analyzed the data with Gene set enrichment analysis using the Molecular Signatures Database (MSigDB) Hallmark gene set collection (Figure 2C). Comparing the patient groups showing the lowest and highest cytokine responses upon *ex vivo* TLR stimulation to the controls, we found 254 and 392 differentially expressed genes (DEGs), respectively (fold change (FC) > 2 or < 0.5, false-discovery rate (FDR) < 0.1, Figure S4). We found that interferon response-related pathways were significantly enriched (FDR < 0.05) in hemodialysis patients from the group showing high cytokine responses versus the controls (Figures 2D and 2E). Interestingly, interferon response-related pathways were also significantly enriched (FDR < 0.05) in hemodialysis patients showing high versus low cytokine responses to TLR stimulation (Figures 2D and 2E), while there was no difference in the interferon response-related gene expression between the group showing low cytokine responses and controls (Figure 2D).

### Trained immunity is mediated by CD4^+^ T cell-derived IFN-γ

The correlation between IFN-γ levels and trained immunity responses in hemodialysis patients suggested that IFN-γ may contribute to a trained immunity phenotype. We previously reported that IFN-γ plays a role in trained immunity responses.^19^

To further explore the involvement of IFN-γ in trained immunity induction, we first examined whether the induction of trained immunity *in vitro* by established training stimuli is accompanied by altered IFN-γ production in PBMCs. For this, we used the well-described trained immunity inducers heat-killed *Candida albicans* (HKCA) and Bacille Calmette Guérin (BCG) vaccine.^20^^,^^21^ We stimulated PBMCs of healthy donors with HKCA or the BCG vaccine for 24 h and quantified IFN-γ production by different cell subsets 24 h, three or six days post-stimulation using flow cytometry. We observed IFN-γ production by CD4^+^ T cells on day six after HKCA and BCG vaccine stimulation (Figures S5 and S6).

**Figure 3:**
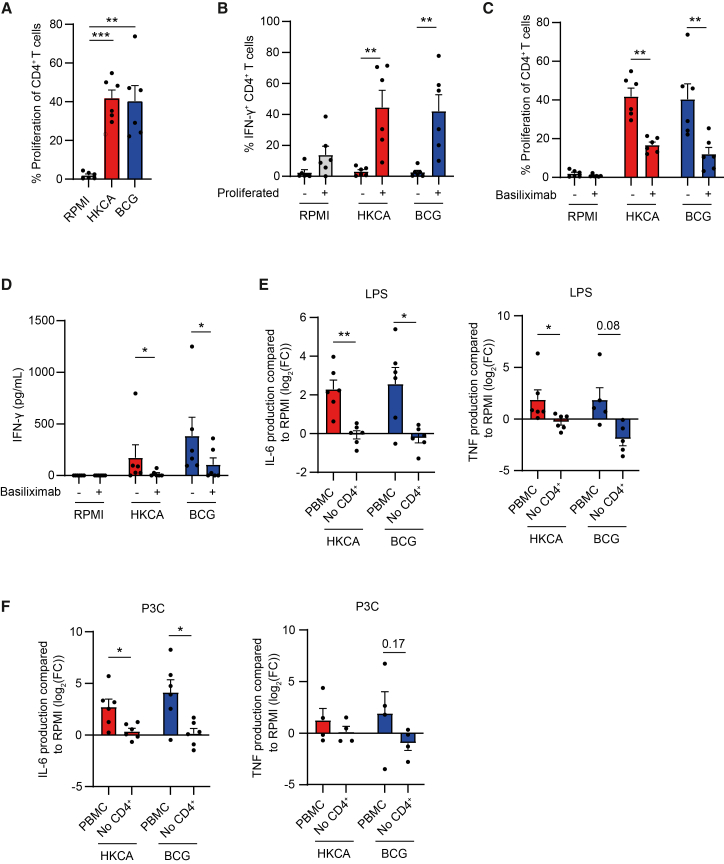
HKCA- and BCG vaccine-induced trained immunity is dependent on IFN-γ production by proliferating CD4^+^ T cells (A) Proliferation of CD4^+^ T cells in co-cultures of monocytes and autologous CFSE-labeled T cells stimulated for 24 h with heat-killed Candida albicans (HKCA), Bacille Calmette Guérin (BCG) vaccine, or RPMI (control), measured 6 days after stimulation (*n* = 6 donors). (B) Interferon gamma (IFN-γ) production in proliferating and non-proliferating CD4^+^ T cells in co-cultures of monocytes and autologous CFSE-labeled T cells stimulated for 24 h with HKCA, BCG vaccine, or RPMI (control), measured 6 days after stimulation (*n* = 6 donors). (C) Proliferation of CD4^+^ T cells in co-cultures of monocytes and autologous CFSE-labeled T cells stimulated for 24 h with HKCA, BCG vaccine, or RPMI (control) in the presence or absence of basiliximab, measured 6 days after stimulation and treatment (*n* = 6 donors). (D) IFN-γ concentrations in supernatant of co-cultures of monocytes and autologous CFSE-labeled T cells stimulated for 24 h with HKCA, BCG vaccine, or RPMI (control) in the presence or absence of basiliximab, measured 6 days after stimulation and treatment (*n* = 6 donors). (E, F) Tumor necrosis factor (TNF) and interleukin 6 (IL-6) production in PBMCs and PBMCs that were depleted from CD4^+^ T cells, stimulated with HKCA or BCG vaccine, compared to untreated cells (RPMI) for 24 h, upon lipopolysaccharide (LPS) (E) or Pam3CSK4 (P3C) (F) restimulation 6 days later (*n* = 6 donors). Mean ± SEM. ∗*p* < 0.05, ∗∗*p* < 0.01, and ∗∗∗*p* < 0.001. Paired one-way ANOVA with Dunnett’s post-test (A) or paired t-tests (B-F) on percentage, log2(fold change), or log10-transformed cytokine concentrations. See also Figures S5 and S6.

To investigate whether IFN-γ production was associated with CD4^+^ T cell proliferation, we prepared co-cultures of monocytes with autologous CFSE-labeled T cells and stimulated the co-cultures with HKCA or BCG vaccine for 24 h. We examined T cell proliferation six days post-stimulation. HKCA and BCG vaccine stimulation increased CD4^+^ T cell proliferation compared to RPMI-treated controls (Figure 3A). The proliferating subset of CD4^+^ T cells showed clear IFN-γ production, while IFN-γ production was absent in non-proliferating CD4^+^ T cells (Figure 3B). To confirm the association between CD4^+^ T cell proliferation and IFN-γ production, we investigated whether the inhibition of CD4^+^ T cell proliferation with basiliximab, a monoclonal antibody targeting the α chain of the IL-2 receptor on T cells, limits IFN-γ production.^22^ Co-cultures of monocytes with autologous CFSE-labeled T cells were stimulated with HKCA, BCG vaccine, or RPMI, and were concomitantly treated with basiliximab for 24 h. We found that basiliximab treatment during stimulation reduced CD4^+^ T cell proliferation and IFN-γ production six days after stimulation with HKCA or the BCG vaccine (Figures 3C and 3D).

Given that proliferating CD4^+^ T cells are the main source of IFN-γ in our trained immunity assay, we wanted to investigate whether CD4^+^ T cell depletion, and the accompanying reduced IFN-γ production, affects the trained immunity response. We stimulated PBMCs and CD4^+^ T cell-depleted PBMCs with RPMI, HKCA, or the BCG vaccine for 24 h. After stimulation, cells were washed and restimulated with LPS or Pam3CSK4 five days later. CD4^+^ T cell depletion prior to the stimulation of PBMCs suppressed HKCA- and BCG vaccine-mediated trained immunity, particularly for the IL-6 response (Figures 3E and 3F).

### IFN-γ enhances pro-inflammatory cytokine production in monocytes *in vitro*

**Figure 4:**
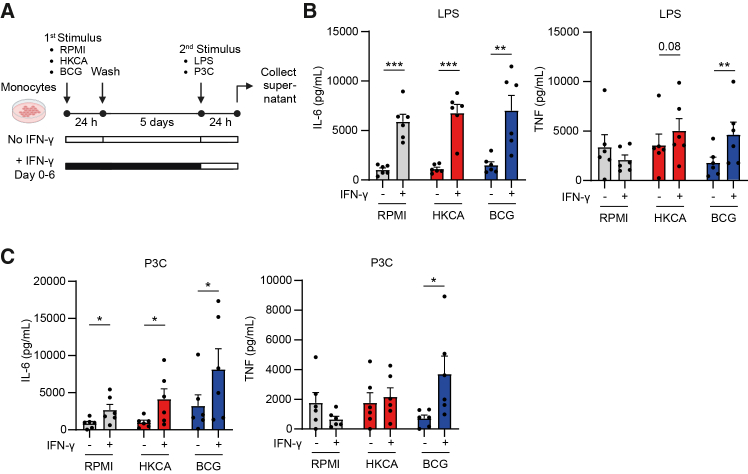
Six-day IFN-γ stimulation enhances cytokine responses in monocytes (A) Schematic representation of *in vitro* trained immunity assays in which purified monocytes were trained heat-killed Candida albicans (HKCA), Bacille Calmette Guérin (BCG) vaccine, or RPMI (control), in combination with 6-day treatment of IFN-γ, and restimulation with lipopolysaccharide (LPS) or Pam3CSK4 (P3C) at day 6. (B, C) Interleukin 6 (IL-6) and tumor necrosis factor (TNF) production upon LPS (B) or Pam3CSK4 (C) restimulation in purified monocytes stimulated with RPMI, HKCA, or BCG vaccine for 24 h, and treated with IFN-γ from day 0 to day 6 of the assay (*n* = 6 donors). Mean ± SEM. ∗*p* < 0.05, ∗∗*p* < 0.01, and ∗∗∗*p* < 0.001; paired t-tests on log10-transfromed cytokine concentrations. See also Figure S7.

As we observed that HKCA- and BCG vaccine-induced trained immunity depended on the presence of CD4^+^ T cells, which produce IFN-γ following HKCA or BCG vaccine stimulation, we further explored the role of IFN-γ in regulating trained immunity. For this purpose, we performed *in vitro* trained immunity experiments in which we stimulated MACS-purified monocytes with HKCA, BCG vaccine, or RPMI for 24 h in the presence or absence of IFN-γ (Figure S7A). TNF and IL-6 production upon LPS or Pam3CSK4 restimulation at day 6 were assessed as a readout for trained immunity induction. We observed that 24-h IFN-γ treatment stimulated IL-6 production upon LPS restimulation in monocytes stimulated with RPMI, HKCA, or BCG vaccine (Figures S7B and S7C). As our previous findings indicated that IFN-γ is gradually produced by CD4^+^ T cells following HKCA or BCG vaccine stimulation in our *in vitro* trained immunity assays, we also tested a protocol in which IFN-γ was applied consistently from day 0 to 6 of the assay (Figure 4A). Administration of IFN-γ for 6 days increased IL-6 production upon LPS or Pam3CKS4 restimulation in monocytes stimulated with HKCA, BCG vaccine or RPMI (Figures 4B and 4C). TNF production was only enhanced in monocytes stimulated with BCG vaccine, but not in those treated with RPMI or HKCA (Figures 4B and 4C).

## Discussion

This study reveals that circulating monocytes of hemodialysis patients display enhanced cytokine responses, particularly for IL-6, to TLR4 and TLR2 stimulation, indicative of a maladaptive trained immunity program. Plasma IFN-γ concentrations were associated with IL-6 and TNF cytokine responses in our LPS stimulation assay. Heightened IL-6 and TNF cytokine responses were also linked to increased expression of interferon-stimulated genes in monocytes, indicating a key role for IFN-γ in shaping the *ex vivo* TLR cytokine responses in hemodialysis patients. To further explore the role of IFN-γ in TLR cytokine responses, we demonstrated that the induction of *in vitro* trained immunity, triggered by the classical trained immunity stimuli HKCA or the BCG vaccine, relies on the presence of IFN-γ-producing CD4^+^ T cells. Collectively, these findings offer new insights into the immune dysregulation in hemodialysis patients, identifying IFN-γ signaling as a potential therapeutic target to mitigate the hyperreactivity of innate immune cells.

Our findings suggest that IFN-γ might play an important role in driving the hyperresponsiveness of innate immune cells in hemodialysis patients, and we observed elevated concentrations of IFN-γ in the plasma of these patients. The source of increased IFN-γ production in hemodialysis patients remains largely unknown. Previous studies identified CD4^+^CD28^null^ T cells—a unique subset of T helper cells with an inherent ability to produce pro-inflammatory cytokines—and the CD56^bright^ NK cell subpopulation in fibrotic kidney tissue as significant sources of IFN-γ.^23^^,^^24^^,^^25^^,^^26^ The triggers that stimulate these cell populations and lead to elevated IFN-γ production are still unclear, though they may be linked to oxidized lipids, uremic toxins, and inflammatory cytokines.^14^ Oxidized low-density lipoprotein (oxLDL), which is increased in hemodialysis patients due to oxidative stress, can promote T cell proliferation and differentiation into IFN-γ-producing Th1 and Th17 cells *in vitro*.^27^^,^^28^^,^^29^^,^^30^ Notably, oxLDL-induced IFN-γ production can be inhibited by MHC class II-blocking antibodies, indicating that this process requires antigen presentation via MHC molecules.^30^ Uric acid, another molecule elevated in the blood of hemodialysis patients, has been found to activate T cells independently of antigenic stimulation.^31^^,^^32^ This activation appears to involve the phosphorylation of the ζ-chain of the T cell receptor and enhanced expression of the transcription factor c-Myc, thereby promoting T cell proliferation.^31^ Besides molecular signals, mechanical stress associated with the dialysis procedure might alter T cell activation and proliferation.^33^ Further research is essential to elucidate the sources and triggers of IFN-γ production in hemodialysis patients.

A potential role for IFN-γ in inducing trained immunity is supported by previous studies. A range of pathogen-related training stimuli, including BCG vaccine, *Candida albicans*, β-glucan, adenoviral vaccines, *P. falciparum*, and the Influenza A virus, have demonstrated involvement of IFN-γ in trained immunity induction in both experimental mouse models and human cell studies.^34^^,^^35^^,^^36^^,^^37^^,^^38^^,^^39^^,^^40^ These studies identified CD4^+^ T cell subsets, CD8^+^ T cells, and NK cells as sources of IFN-γ, depending on the experimental conditions (e.g., type of stimulus and route of administration). The production of IFN-γ by T cells following stimulation with a training stimulus required direct, CD40-CD40L-mediated interactions with monocytes.^19^ Addition of IFN-γ to the *in vitro* training of purified monocytes can enhance cytokine production overall, but cannot reproduce the hyperresponsive cytokine production of monocytes trained in the presence of T cells. The role of IFN-γ and T cells in inducing trained immunity in a non-infectious context, such as in our cohort of hemodialysis patients, has not been previously documented.

Previous studies have indicated that IFN-γ-induced trained immunity operates through JAK-STAT signaling pathways. Treatment with the STAT-inhibitors ruxolitinib and tofacitinib suppressed trained immunity induced by BCG vaccine or *P. falciparum*-infected red blood cells.^38^^,^^41^ Qiao et al. found that IFN-γ stimulation leads to the sustained presence of the transcription factors STAT1 and IRF-1 at the TNF and IL-6 loci, which recruit histone acetyltransferases (HATs) to these regions.^42^ Modulation of histone marks at these loci may play a crucial role in establishing the chromatin signatures characteristic of trained immunity.^43^^,^^44^ Besides epigenetic modulation, IFN-γ stimulation was shown to enhance TLR4 expression, which might contribute to the exaggerated responsiveness of monocytes observed upon LPS restimulation.^45^

We observed a time-dependent relationship between the duration of IFN-γ stimulation and the strength of the trained immunity-induced response. This is consistent with findings by Crabtree et al. and Jacobs et al., who reported that 4-day or 6-day incubation with IFN-γ led to increased cytokine responses upon subsequent TLR stimulation *in vitro* compared to untreated controls.^19^^,^^38^ The finding that long-term, and to a lesser extent 1-day stimulation with IFN-γ, enhances cytokine responses is corroborated by *in vivo* trained immunity models, in which the proliferation of IFN-γ producing T cell subsets and sustained IFN-γ production was observed only a few days post-stimulation.^34^^,^^35^ How the duration and timing of IFN-γ stimulation impact the epigenome needs to be further investigated.

Collectively, our study reveals that circulating monocytes in hemodialysis patients exhibit enhanced cytokine responses to TLR4 and TLR2 stimulation, suggesting the induction of a maladaptive trained immunity phenotype. A strong correlation between monocyte hyperresponsiveness and plasma IFN-γ levels indicates a crucial role of this cytokine in these responses. Our *in vitro* trained immunity model further demonstrated that IFN-γ produced by CD4^+^ T cells plays an important role in inducing innate immune memory. These findings suggest that therapeutically modulating IFN-γ signaling may be an effective strategy for mitigating immune hyperreactivity in hemodialysis patients.

### Limitations of the study

We investigated the role of trained immunity and innate immune cell hyperresponsiveness in hemodialysis patients. We found that pro-inflammatory cytokine responses of PBMCs from hemodialysis patients correlate with plasma levels of IFN-γ as measured using Olink proteomics. The Olink panel used for our studies measures 92 proteins in a pre-designed inflammation panel. Therefore, we cannot exclude that other protein markers not measured in the panel could be associated with the hyperresponsiveness of innate immune cells. Future studies should investigate this in an unbiased manner. We showed that CD4^+^ T cells are an important source of IFN-γ in our trained immunity assay. However, we cannot exclude that other cell types (e.g., NK cells) also produce IFN-γ that might be relevant in our assay. Since CD4^+^ T cells are the most abundant IFN-γ-producing cells, we think that the role of other cells will be minimal. One potential technical limitation is that we cannot exclude that in our trained immunity assay, the initial stimulus remains present in the culture even after the extensive washing step after 24 h. Since we do not have any information about the sex or gender of our blood donors from the blood bank from which we used the PBMCs for our *in vitro* trained immunity assays, we cannot analyze the effect of sex and age on the responses measured.

## Resource availability

### Lead contact

Requests for further information and resources should be directed to and will be fulfilled by the lead contact, Raphael Duivenvoorden (raphael.duivenvoorden@radboudumc.nl).

### Materials availability

This study did not generate new unique reagents.

### Data and code availability

• RNA sequencing data have been deposited at GEO: GSE290317 and are publicly available as of the date of publication.
• This paper does not report original code.
• Any additional information required to reanalyze the data reported in this paper is available from the lead contact upon request.

## Acknowledgments

This work was supported by the Hypatia grant by the 10.13039/501100006209Radboud University Medical Center, the 10.13039/100000002National Institutes of Health (1P01AI168258-01A1, to J.O., L.A.B.J., A.J.P.T., M.G.N., and R.D.), and the Senior Kolff grant by the Dutch Kidney Foundation (to R.D.). M.G.N. and L.A.B.J. are supported by the Dutch 10.13039/100002129Heart Foundation IN-CONTROL CVON grant [CVON2018-27]. M.G.N. was supported by an ERC advanced Grant (#8333247) and a Spinoza Grant of the 10.13039/501100003246Netherlands Organisation for Scientific Research.

## Author contributions

Conceptualization, I.J., M.M.E.J., N.R., and R.D.; methodology, I.J., M.M.E.J., L.S.H., Y.N., N.R., and R.D.; investigation, I.J., M.M.E.J., L.S.H., and N.R.; writing – original draft, I.J. and M.M.E.J.; writing – review and editing, I.J., M.M.E.J., L.S.H., J.O., J.M., M.M., A.T., L.J., M.N., L.B.H., N.R., and R.D.; funding acquisition, R.D.; supervision, J.O., J.M., M.M., A.T., L.J., M.N., L.B.H., N.R., and R.D.

## Declaration of interests

J.O., L.A.B.J., and M.G.N. declare that they are scientific founders of Trained Therapeutics Discovery. The other authors declare no competing interests.

## STAR★Methods

### Key resources table

**Table:** 

REAGENT or RESOURCE	SOURCE	IDENTIFIER
**Antibodies**		

Anti-human CD45-BV510	Biolegend	Cat#304035; RRID: AB_2561383
Anti-human CD56-APC	Beckman-Coulter	Cat#IM2474; RRID: AB_130791
Anti-human CD3-APC/Cy7	Biolegend	Cat#317342; RRID: AB_2563410
Anti-human CD19-PE/Cyanine5	Biolegend	Cat#302210; RRID: AB_314240
Anti-human CD16-FITC	eBioscience	Cat#11-0168-42; RRID: AB_10805747
Anti-human CD14-PE/Cyanine7	eBioscience	Cat#25-0149-42; RRID: AB_1582276
Anti-human CD11b-BV785	Biolegend	Cat#301346; RRID: AB_2563794
Anti-human CCR2-BV421	BD Biosciences	Cat#564067; RRID: AB_2738573
Anti-human HLA-DR-PE	Beckamn-Coulter	Cat#IM1639; RRID: AB_131284
Anti-human CX3CR1-BV650	Biolegend	Cat#341626; RRID: AB_2716245
Anti-human CD4-BV650	Biolegend	Cat#344691; RRID: AB_2936743
Anti-human CD19-ECD	Beckman-Coulter	Cat#6604551; RRID: AB_1575950
Anti-human CD8-PerCP	Biolegend	Cat#980916; RRID: AB_2890877
Anti-human CD3-APC	Beckman Coulter	Cat#IM2467; RRID: AB_130788
Anti-human IFN-γ PE/Cyanine-7	Biolegend	Cat#506518; RRID: AB_2123321

**Biological samples**		

Human Buffy coats	Sanquin Nijmegen	Cat#B2825R00

**Chemicals, peptides, and recombinant proteins**		

Ficoll-Paque	Lymphoprep, Stem cell Technologies	Cat#07861
RPMI 1640	Thermo Fisher Scientific	Cat#22409
Glutamax	Thermo Fisher Scientific	Cat#35050
Pyruvate	Thermo Fisher Scientific	Cat#11360
Penicillin/Streptomycin	Thermo Fisher Scientific	Cat#15140
Fixable Viability stain 620	BD Biosciences	Cat#564996
Bovine Serum Albumin	Sigma	Cat#A3803
Heat-inactivated human serum	Serana	Cat#S-HU-EU-011
Brilliant Stain Buffer	BD Biosciences	Cat#563794
Ultrapure Lipopolysaccheride (E.coli, O111:B4)	Invivogen	Cat#tlrl-3pelps
Pam3CSK4	Invivogen	Cat#tlrl-pms
RNA later Buffer	Qiagen	Cat#79216
DNaseI	Qiagen	Cat#79254
Heat-killed Candida albicans	Invivogen	Cat#tlrl-hkca
Bacille Calmette-Guérin Vaccine	Intervax	Bulgaria strain
Basiliximab (Simulect 10 mg)	Novartis	N/A
EDTA	Sigma	Cat#E5134
Fixation/Permeabilization Solution Kit with BD GolgiPlug™	BD Biosciences	Cat#555028
LIVE/DEAD™ Fixable Near-IR Dead Cell Stain	Invitrogen	Cat#L34975
Zombie Violet Fixable Viability Stain	Biolegend	Cat#423107

**Critical commercial assays**		

Human TNF-α DuoSet ELISA	R&D systems	Cat#DY210
Human IL-6 DuoSet ELISA	R&D systems	Cat#DY206
Human IFN-gamma DuoSet ELISA	R&D systems	Cat#DY285B
Olink Target 96 Inflammation panel	Olink Bioscience	N/A
RNeasy kit	Qiagen	Cat#74182
Human Pan T cell Isolation Kit	Miltenyi Biotec	Cat#130-096-535
Human Pan Monocyte Isolation Kit	Miltenyi Biotec	Cat#130-096-537
CellTrace™ CFSE Cell Proliferation Kit	Thermo Fisher Scientific	Cat#C34554
Human CD4^+^ T cell isolation kit	Miltenyi Biotec	Cat#130-096-533
Recombinant human IFN-γ protein	Invivogen	Cat#rcyec-hinfg

**Deposited data**		

RNA sequencing data	This paper	GSE290317

**Software and algorithms**		

FlowJo^TM^ v10	BD Biosciences	N/A
R studio	R Core Team	https://www.r-project.org/
DESeq2	Love et al.^46^	https://www.bioconductor.org/packages/release/bioc/html/DESeq2.html
GSEA v4.1.0	Subramanian et al.^47^	https://www.gsea-msigdb.org/gsea/index.jsp
Molecular Signature database	Liberzon et al.^48^	https://www.gsea-msigdb.org/gsea/msigdb/index.jsp
SPSS v29.0	IBM	N/A
Prism	GraphPad	N/A

**Other**		

Sysmex XN-450	Sysmex	N/A
ACEA Novocyte 3000	Agilent	N/A

### Experimental model and study participant details

#### Studies in samples of hemodialysis patients and healthy controls

We studied five healthy individuals and twenty-six patients treated with chronic intermittent hemodialysis at the Radboud University Medical Center, a tertiary care university hospital in the Netherlands. Only healthy controls and hemodialysis patients of 18 years and older were included and all provided written informed consent before study entry. The study was approved by the Committee on Human-Related Research Arnhem-Nijmegen (#2019-5115) and conducted according to the Declaration of Helsinki and good clinical practice guidelines. Blood was obtained just prior to the start of the patient’s hemodialysis session.

#### *In vitro* studies on human PBMCs

For *in vitro* studies on human PBMCs, buffy coats from healthy donors were obtained from Sanquin blood bank, Nijmegen after obtaining written consent. No further information about donor sex, age or other characteristics are known.

### Method details

#### PBMC isolation

PBMCs were isolated by differential centrifugation over Ficoll-Pague (Lymphoprep, Stemcell Technologies). Cells were washed three times in PBS, and PBMCs were resuspended in RPMI 1640 culture medium supplemented with 2 mM glutamax, 1 mM pyruvate, and penicillin/streptomycin (Thermo Fisher Scientific) and counted on a Casy cell counter or Sysmex XN-450 automated hematology analyzer.

#### Flow cytometry on PBMCs of HD patients and healthy controls

PBMCs from HD patients and healthy controls (500.000 cells/sample) were stained with Fixable Viability Stain 620 (BD Biosciences) for 15 min at room temperature, according to manufacturer’s instructions. Cells were washed 2 times with PBS containing 1 % bovine serum albumin (BSA) and blocked in 10 % heat-inactivated human serum for 30 min on ice in the dark. Cells were stained for extracellular markers for 30 min on ice in dark using the following antibodies: anti-human CD45-BV510 (Biolegend, 1:100, RRID: AB_2561383), anti-human CD56-APC (Beckman Coulter, 1:50, RRID:AB_130791), anti-human CD3-APCCy7 (Biolegend, 1:100, RRID: AB_2563410), anti-human CD19-PE/Cyanine5 (Biolegend, 1:100, RRID:AB_314240), anti-human CD16-FITC (eBioscience, 1:100, RRID: AB_10805747), anti-human CD14-PE/Cyanine7 (eBioscience, 1:100, RRID:AB_1582276), anti-human CD11b- BV785 (Biolegend, 1:100, RRID:AB_2563794), anti-human CCR2-BV421 (BD Biosciences, 1:50, RRID:AB_2738573), anti-human HLA-DR-PE (Beckman Coulter, 1:20, RRID:AB_131284), and anti-human CX3CR1-BV650 (Biolegend, 1:50, RRID:AB_2716245) in presence of 10 % Brilliant Stain Buffer (BD Biosciences). Cells were washed 2 times with PBS containing 1 % BSA and were resuspended in PBS 1 % BSA for acquisition. Acquisition was performed using an ACEA Novocyte 3000 (Agilent). Analyses and preparation of tSNE plots were performed in FlowJo v10 Software (BD Biosciences).

#### Lipopolysaccharide and Pam3CSK4 *ex vivo* stimulations

PBMCs from HD patients and healthy controls were plated in 96-well flat bottom plates, with a density of 500.000 cells per well. Cells were left to adhere for one hour at 37°C at 5% CO2 (v/v). After one hour cells were washed 3 times with PBS and incubated with RPMI culture medium only (negative control), 10 ng/mL lipopolysaccharide (LPS) (InvivoGen, Ultrapure (E.coli, O111:B4)), or 1 μg/mL Pam3CSK4 (P3C) (InvivoGen) for 24 hours. After 24 hours supernatant was collected.

#### Cytokine/protein measurements

Cytokine production in supernatants was measured using commercial ELISA kits for human TNF, IFN-γ and IL-6 (R&D systems) according to the manufacturer’s instruction.

#### Measurement of inflammatory proteins in plasma

Inflammation-related protein biomarkers were measured by Olink Proteomics, with the Olink Proximity Extension Assay (PEA) technology (Olink Target 96 Inflammation panel, Olink Bioscience, Sweden). Protein levels are shown in Normalized Protein eXpression (NPX) values on a log2 scale. Proteins were excluded when over 50% of the samples were under the limit of detection (LOD).

#### RNA isolation, library preparation and sequencing for transcriptomic analysis

For RNA isolation, 1∗10^6^ monocytes were resuspended in 350 μL of RNA later Buffer (QIAGEN). RNA was isolated using RNeasy kit (QIAGEN) including deoxyribonuclease (DNase)I (QIAGEN) digestions. RNA bulk sequencing was performed by Single Cell Discoveries (Utrecht, The Netherlands) with a sequencing depth of 20∗10^6^ reads/sample. Library preparation was performed according to the CEL-seq2 protocol.^49^ Sequencing was performed on a Nextseq500 (Illumina).

#### Monocyte and T cell isolation

Monocytes and T cells were isolated from PBMCs by negative MACS isolation using the Pan Monocyte Isolation Kit and the Pan T Cell Isolation Kit (Miltenyi Biotec) according to manufacturer’s instructions. Cell numbers and purity were measured with a Sysmex XN-450 automated hematology analyzer. Purity reached >90% and 95-99% for monocytes and T cells respectively.

#### T cell proliferation assay in autologous monocyte:T cell co-cultures

Purified monocytes were seeded in 12 well plates in a density of 1∗10^6^ cells/mL and allowed to adhere for 1 hour. Purified T cells were labeled with CFSE using the CellTrace™ CFSE Cell Proliferation Kit (Thermo Fisher Scientific) (1 μM for 20 min). Cells were washed with culture medium containing 10 % FBS and counted. CFSE-labelled T cells were seeded in a density of 2∗10^6^ cells/mL per well. Co-cultures were stimulated with RPMI culture medium (negative control), HKCA (10^5^ cells/mL) or BCG vaccine (5 μg/mL) for 24 hours. Where indicated, basiliximab (10 μg/mL, Novartis) was added during stimulation for 24 hours. We previously determined that 24 hour basiliximab treatment did not have cytotoxic effects.^50^ Subsequently, cells were washed 3 times with PBS and rested for 5 days in RPMI culture medium containing 10 % FBS. At day 6, cells were washed 2 times with PBS and incubated in Versene solution (0.48 mM EDTA in PBS) for 30 min at 37°C, 5 % CO2 (v/v). Cells were scraped, counted and stained for flow cytometric analysis. CFSE dilution was quantified to measure T cell proliferation.

#### Flow cytometry on PBMCs from buffy coats and monocyte:T cell co-cultures

PBMCs and monocyte:T cell co-cultures were stimulated as described previously and harvested at 24 hours, 3 days or 6 days post-stimulation. For harvesting, cells were incubated in 0.48 mM EDTA in PBS for 30 min at 37°C, 5 % CO2 (v/v). Cells were scraped, counted and seeded in a 96-wells V-bottom plate. Cells were incubated in RPMI culture medium containing 10 % FBS with GolgiPlug protein transport inhibitor (BD Biosciences) for 4 - 5 hours. Cells were stained with LIVE/DEAD™ Fixable Near-IR Dead Cell Stain (Invitrogen) or Zombie Violet Fixable Viability Stain (Biolegend) according to manufacturer’s instructions. Cells were washed 2 times with PBS containing 1 % BSA and blocked in 10 % heat-inactivated human serum for 30 min on ice in dark. Cells were stained for extracellular markers for 30 min on ice in the dark and in the presence of 10 % Brilliant Stain Buffer. Antibodies used were anti-human CD45- BV510 (Biolegend, 1:100, RRID: AB_2561383), anti-human CD16-FITC (eBioscience, 1:100, RRID: AB_10805747), anti-human CD3-APCCy7 (Biolegend, 1:100, RRID: AB_2563410), anti-human CD11b-BV785 (Biolegend, 1:100, RRID:AB_2563794), anti-human CD4 BV650 (Biolegend, 1:100, RRID: AB_2936743), anti-human CD19-ECD (Beckman Coulter, 1:100, RRID:AB_2940901), anti-human CD56-APC (Beckman Coulter, 1:50, RRID:AB_130791), anti-human CD8-PerCP (Biolegend, 1:100, RRID: AB_2890877), anti-human CD3-APC (Beckman Coulter, 1:100, RRID: AB_130788). Cells were washed 2 times with PBS containing 1 % BSA. For intracellular IFN-γ staining, cells were fixated and permeabilized using Fixation/Permeabilization Solution Kit (BD Biosciences) according to manufacturer’s instructions. Cells were incubated with anti-human IFN-γ PE/Cyanine-7 (Biolegend, 1:50, RRID:AB_2123321) for 30 min on ice in the dark. Cells were washed 2 times with PBS containing 1 % BSA and were resuspended in PBS 1 % BSA for acquisition. Acquisition was performed using an ACEA Novocyte 3000 (Agilent). Analyses were performed in FlowJo v10 Software (BD Biosciences).

#### *In vitro* trained immunity assays with CD4^+^ T cell-depleted PBMCs

CD4^+^ T cells were depleted from PBMCs by performing two cycles of positive CD4^+^ T cell isolation using the human CD4^+^ T cell isolation kit (Miltenyi Biotec) according to the manufacturer’s instructions. Flowthrough was collected and cells were counted. Depletion efficiency was validated using flow cytometry. PBMCs contained < 1 % CD4^+^ T cells after depletion. For performing trained immunity assays, seeding density of full PBMCs and CD4^+^ T cell-depleted PBMCs was normalized for monocyte percentage. Cells were seeded in 96-wells flat bottom plates with a density of 100.000 monocytes per well and allowed to adhere for 1 hour. Subsequently, trained immunity assays were performed as described previously.

#### *In vitro* trained immunity assays

Human PBMCs or MACS-purified monocytes were plated in 96-well flat bottom plates, with a density of 500.000 or 100.000 cells per well respectively. Cells were left to adhere for one hour at 37°C at 5% CO2 (v/v). After one hour cells were washed 3 times with PBS. After washing PBMCs were incubated with RPMI culture medium only (negative control), heat-killed Candida Albicans (HKCA) (10^5^ cells/mL, InvivoGen), BCG vaccine (5 μg/mL, BCG-Bulgaria, Intervax), or human recombinant IFN-γ (50 ng/mL, InvivoGen) for 24 hours at 37°C. After 24 hours cells were washed and rested for five days in culture medium containing 10 % FBS. Where indicated, IFN-γ stimulation (50 ng/mL) was continued up to day 6 of the trained immunity assay. At day 6, cells were stimulated with RPMI culture medium (negative control), 10 ng/mL LPS (InvivoGen) or 1 μg/mL Pam3CSK4 (InvivoGen) for 24 hours.

### Quantification and statistical analysis

#### RNA-seq data analyses

Differential gene expression analysis was performed on counts of 10 patients and 5 healthy controls using the DESeq2 package in Rstudio.^46^ Differentially Expressed Genes (DEGs) were defined as fold change (FC) < 0.5 or FC > 2 and FDR < 0.1.^51^

#### Gene set enrichment analysis

Gene set enrichment analyses (GSEA) were performed on normalized counts of samples using GSEA software v4.1.0 provided by Broad Institute.^47^ GSEA was performed using the gene sets from HALLMARK database of the Molecular Signature database (MSigDB).^48^ Analyses were conducted with 1000 gene set permutations and with the following settings: Metric for ranking genes: Signal2Noise; Remap/Collapse to gene symbols: Collapse; Enrichment statistic: weighted; Normalization mode: meandiv. For each gene set, a Normalized Enrichment Score (NES) was calculated. Gene sets for which FDR < 0.1 were considered to be enriched.

#### Statistical analysis

Continuous data are presented as mean with standard deviation (SD) or standard error of the mean (SEM). In case of non-normal distribution of the data, medians and interquartile ranges are reported. Categorical data are presented as percentages. Cytokine data were Log10-transformed. Unpaired t test, paired t test, or 1-way analysis of variance (ANOVA) with Dunnett’s post-test were used to assess between group differences. A p value < 0.05 was deemed statistically significant. Spearman correlations were used to assess correlations between plasma markers of inflammation and trained immunity cytokine responses. Multivariable regression models were obtained using a backward elimination procedure. Variables were retained if p < 0.1. Analyses were performed with the statistical software IBM SPSS Statistics for Windows, Version 29.0 IBM Corp and GraphPad Prism 9.1.2. Statistical details including statistical test, exact value of n and what n represents can be found in the figure legends.
