# Supplementary material for: Immune hyperactivity in hemodialysis patients is associated with interferon gamma-induced trained immunity
Source: iScience. 2026 Jun 23;29(7):116496. doi: 10.1016/j.isci.2026.116496 (PMC13320352; doi:10.1016/j.isci.2026.116496)
Supplement: Document S1. Figures S1–S7 and Tables S1–S6 [file mmc1.pdf]

iScience, Volume *29*

Supplemental information

Immune hyperactivity in hemodialysis patients is associated with interferon gamma-induced trained immunity

Inge Jonkman, Maaike M.E. Jacobs, Leonie S. Helder, Yutaka Negishi, Jordi Ochando, Joren C. Madsen, Musa M. Mhlanga, Abraham J.P. Teunissen, Leo A.B. Joosten, Mihai G. Netea, Luuk B. Hilbrands, Nils Rother, and Raphaël Duivenvoorden

Supplementary Material

This article contains the following supplemental material:

- Supplementary Table S1. Demographics and characteristics of healthy controls.
- Supplementary Table S2. Results of multivariate linear regression analysis with backward elimination of clinical parameters and patients' cell IL-6 and TNF response after LPS or Pam3CSK4 stimulation.
- Supplementary Table S3. Results of multivariate linear regression analysis with backward elimination of clinical parameters and IFN-γ levels
- Supplementary Table S4. Results of multivariate linear regression analysis with backward elimination of clinical parameters and IFN-γ and patients' cell IL-6 and TNF response after LPS or Pam3CSK4 stimulation.
- Supplementary Table S5. Correlation of Olink proteomic results with dialysis duration and Charlson Comorbidity Index.
- Supplementary Table S6. Demographics and characteristics of dialysis patients used for RNA-seq analysis.
- Supplementary Figure S1: Immune cell profile and response of hemodialysis patients and healthy controls.
- Supplementary Figure S2: Immune cell marker expression on monocytes.
- Supplementary Figure S3: No significant correlation between percentage of monocytes in PBMCs and IL-6 or TNF cytokine responses
- Supplementary Figure S4: Effects of hemodialysis on transcriptional profiles of monocytes compared to healthy controls.
- Supplementary Figure S5: Gating strategy for determining proliferation and IFN-γ production in T cells using flow cytometry
- Supplementary Figure S6: Production of IFN-γ following stimulation with HKCA, BCG and RPMI
- Supplementary Figure S7: Short-term IFN-γ stimulation enhances LPS-induced IL-6 production in monocytes.

Supplementary Table S1. Demographics and characteristics of healthy controls.

Characteristics	N=5
Age, years	59 (57.5 – 65.0)
Male, no. (%)	1 (20%)

Data are presented as the median (interquartile range) or number (no.) and percentage.

Supplementary Table S2. Results of multivariate linear regression analysis with backward elimination of clinical parameters and patients' cell IL-6 and TNF response after LPS or Pam3CSK4 stimulation.

Characteristics	B	95% Confidence Interval	p-value
**<u>LPS</u>**			
**IL-6**			
Percentage of monocytes	801.731	-80.725 – 1684.187	0.073
Immunosuppressants	-25575.684	-49484.267 – -1667.101	0.037
**TNF**			
Immunosuppressants	-2164.696	-4471.500 – 142.109	0.065
**<u>Pam3CSK4</u>**			
**IL-6**			
Immunosuppressants	-18123.51	-39881.81 – 3634.794	0.098
**TNF**			
-			

Parameters included; Age, Sex, Pre-dialysis serum urea level, Kt/V, Albumin, CRP, Charlson Comorbidity Index, Immunosuppressant use and monocyte percentage. IL-6: interleukin 6; LPS: lipopolysaccharide; TNF: tumor necrosis factor.

Supplementary Table S3. Results of multivariate linear regression analysis with backward elimination of clinical parameters and IFN-γ levels

Characteristics	B	95% Confidence Interval	p-value
**IFN-γ**			
Sex	0.858	-0.118 – 1.834	0.082
Kt/V	1.866	0.371 – 3.361	0.017

Parameters included; Age, Sex, Pre-dialysis serum urea level, Kt/V, Albumin, CRP, Charlson Comorbidity Index, Immunosuppressant use. IFN-γ: interferon gamma.

Supplementary Table S4. Results of multivariate linear regression analysis with backward elimination of clinical parameters and IFN-γ and patients' cell IL-6 and TNF response after LPS or Pam3CSK4 stimulation.

Characteristics	B	95% Confidence Interval	p-value
**<u>LPS</u>**			
**IL-6**			
Age	828.416	-5.956 – 1662.787	0.052
Sex	-24065.494	-49069.109 – 938.121	0.058
IFN-γ	11050.530	1132.222 – 20968.838	0.031
**TNF**			
Charlson Comorbidity Index	627.159	51.318 – 1203.000	0.034
Immunosuppressants	-1735.780	-3335.936 – 135.624	0.035
Albumin	157.718	-18.852 – 334.287	0.077
IFN-γ	1938.966	1197.063 – 2680.870	<0.001
**<u>Pam3CSK4</u>**			
**IL-6**			
-			
**TNF**			
Charlson Comorbidity Index	380.194	64.894 – 695.493	0.020
Immunosuppressants	-809.931	-1738.624 – 118.762	0.084
IFN-γ	1244.451	818.575 – 1670.327	<0.001

Parameters included; Age, Sex, Pre-dialysis serum urea level, Kt/V, Albumin, CRP, Charlson Comorbidity Index, Immunosuppressant use, IFN-γ. IFN-γ: interferon gamma; IL-6: interleukin 6; LPS: lipopolysaccharide; TNF: tumor necrosis factor.

Supplementary Table S5. Correlation of Olink proteomic results with dialysis duration and Charlson Comorbidity Index.

Characteristics	Spearman rho	p-value	Adjusted p-value
**Dialysis duration**			
HGF	0.704	6.05e-5	6.60e-3
**CCI**			
uPA	0.739	1.61e-5	1.75e-3
TNFSF14	0.663	2.24e-4	9.29e-3
CDCP1	0.658	2.56e-4	9.29e-3

HGF: Hepatocyte growth factor; CCI: Charlson Comorbidity Index; uPA: Urokinase; TNFSF14: Tomur necrosis factor super family member 14; CDCP1: CUB Domain-Containing Protein 1. Multiple testing correction: Benjamini-Hochberg.

Supplementary Table S6. Demographics and characteristics of dialysis patients used for RNA-seq analysis.

Characteristics	Total<br>N=10	Low<br>N=5	High<br>N=5
Age, years	67.5 (61.0 – 73.5)	62.0 (60.5 – 71.5)	70.0 (64.0 – 75.5)
Male, no. (%)	6 (60%)	4 (80%)	2 (40%)
BMI	24.7 (21.9 – 27.0)	23.7 (21.9 – 26.3)	26.2 (21.3 – 37.9)
Systolic blood pressure (mm Hg)	146 (23.8)	138 (27.5)	153 (19.7)
Diastolic blood pressure (mm Hg)	70 (14.4)	72 (17.7)	66 (11.3)
Hospital admissions last year	1 (0.8 – 2)	1 (1 – 2)	1 (0 – 2.5)
Charlson Comorbidity Index	6 (1.2)	6 (1.0)	7 (1.3)
**History**			
Cardiovascular disease, no. (%)	8 (80%)	3 (60%)	5 (100%)
Diabetes, no. (%)	4 (40%)	1 (20%)	3 (60%)
Glomerulonephritis or autoimmune disease, no. (%)	1 (10%)	1 (20%)	0 (0%)
Cancer, no. (%)	1 (10%)	0 (0%)	1 (20%)
Previous kidney transplant, no. (%)	3 (30%)	1 (20%)	2 (40%)
**Medication use**			
Immunosuppressant use, no. (%)	5 (50%)	4 (80%)	1 (20%)
Corticosteroids, no. (%)	5 (50%)	4 (80%)	1 (20%)
ACEi/ARB, no. (%)	2 (20%)	1 (20%)	1 (20%)

Statin, no. (%)	3 (30%)	0 (0%)	3 (60%)
Glucose lowering drugs, no. (%)	3 (30%)	1 (20%)	2 (40%)
Insulin, no. (%)	3 (30%)	1 (20%)	2 (40%)
Platelet aggregation inhibitor, no. (%)	4 (40%)	1 (20%)	3 (60%)
Vitamin K antagonist or DOAC, no. (%)	1 (10%)	0 (0%)	1 (20%)
**Dialysis related parameters**			
Duration on dialysis (months)	31.0 (6.8 – 152.8)	7.0 (6.0 – 172.0)	70.0 (28.0 – 177.5)
Frequency of dialysis	3 (3 – 3)	3 (3 – 3)	3 (3 – 3)
Kt/V	1.39 (0.35)	1.21 (0.35)	1.56 (0.28)
Hemodialysis vascular access, no. (%)			
Arteriovenous graft	1 (10%)	1 (20%)	0 (0%)
Arteriovenous fistula	4 (40%)	2 (40%)	2 (40%)
Catheter	5 (50%)	2 (40%)	3 (60%)
Estimated residual diuresis	376.0 (0.0 – 739.5)	600.0 (260.0 – 1753.5)	0.0 (0.0 – 376.0)
Mean	537.9	925.4	150.4
**Lab values**			
Albumin (mg/mmol)	32.7 (5.6)	31.0 (7.6)	34.4 (2.5)
Ureum (mg/dL)	24.5 (20.6 – 30.4)	30.2 (18.6 – 71.5)	22.8 (20.3 – 27.6)
C-reactive protein (mg/L)	6.0 (2.0 – 7.3)	4.0 (2.0 – 7.5)	7.0 (3.0 – 10.5)
PTH (pg/mL)	16.5 (7.9 -26.3)	8.2 (7.0 – 18.5)	20.0 (16.0 – 45.0)
iCa (mmol/L)	1.15 (1.13 – 1.20)	1.18 (1.14 – 1.21)	1.13 (1.11 – 1.20)

Potassium (mEq/L)	4.6 (0.5)	4.7 (0.6)	4.5 (0.4)
Sodium (mmol/L)	135.1 (3.2)	134.6 (1.9)	135.6 (4.3)
pH	7.36 (0.03)	7.37 (0.03)	7.35 (0.02)
Bicarbonate (mmol/L)	23.9 (2.0)	24.9 (1.4)	22.8 (2.0)
Phosphorus (mmol/L)	1.37 (1.26 – 1.57)	1.32 (1.30 – 1.52)	1.42 (1.16 – 1.65)
Hb (mmol/L)	6.8 (0.6)	7.0 (0.5)	6.5 (0.5)
Leukocytes (10^9/L)	8.3 (7.0 – 9.1)	8.2 (5.7 – 9.0)	8.3 (7.1 – 10.0)
Thrombocytes (10^9/L)	260.0 (67.4)	271.2 (92.3)	248.8 (37.4)

Data are presented as the mean (SD) for normalized data, median (interquartile range) for non-normalized data, or number (no.) and percentage. ACEi: angiotensin-converting enzyme inhibitors; ARB: angiotensin II receptor blockers; BMI: body mass index; DOAC: direct oral anticoagulants; Hb; hemoglobin; iCa: ionized calcium; PTH: Parathyroid hormone.

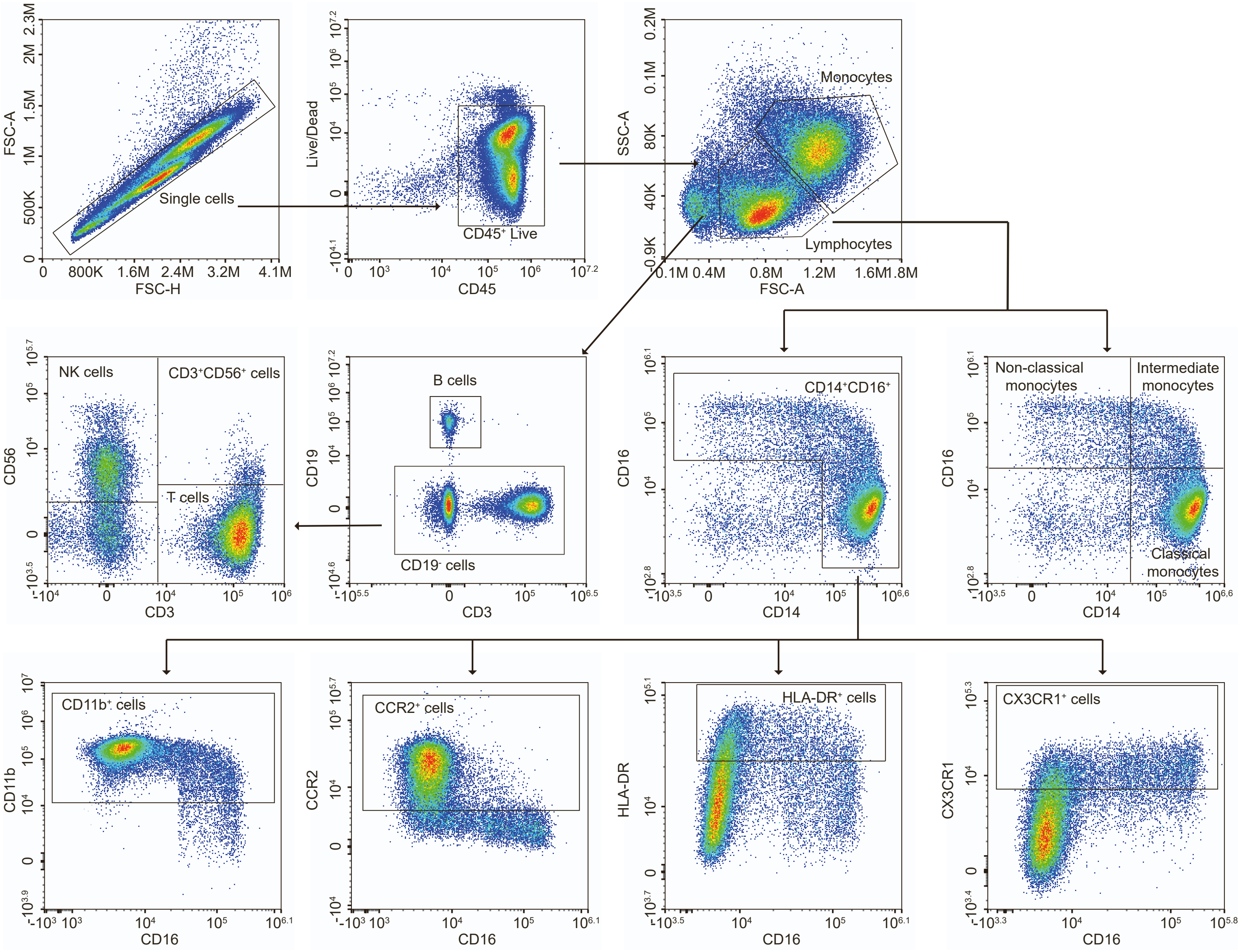

Figure S1: Immune cell profile and response of hemodialysis patients and healthy controls. Flow cytometry gating strategy for quantifying lymphocytes, monocytes and marker expression on monocytes.

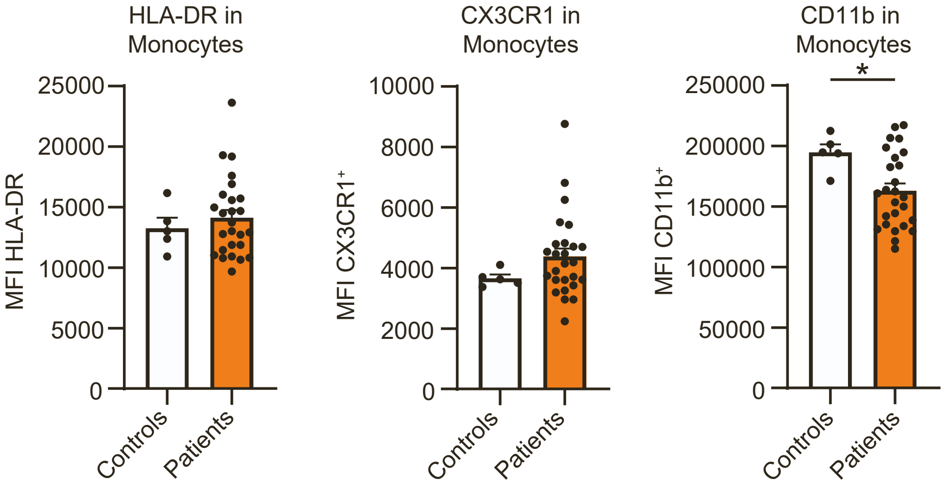

Figure S2: Immune cell marker expression on monocytes.
Quantification of HLA-DR, CX3CR1, and CD11b marker expression on monocytes of hemodialysis (HD) patients and healthy controls.
Mean ± SEM. (n=5 healthy controls, n=26 HD patients) * $p < 0.05$. Two-tailed t tests.

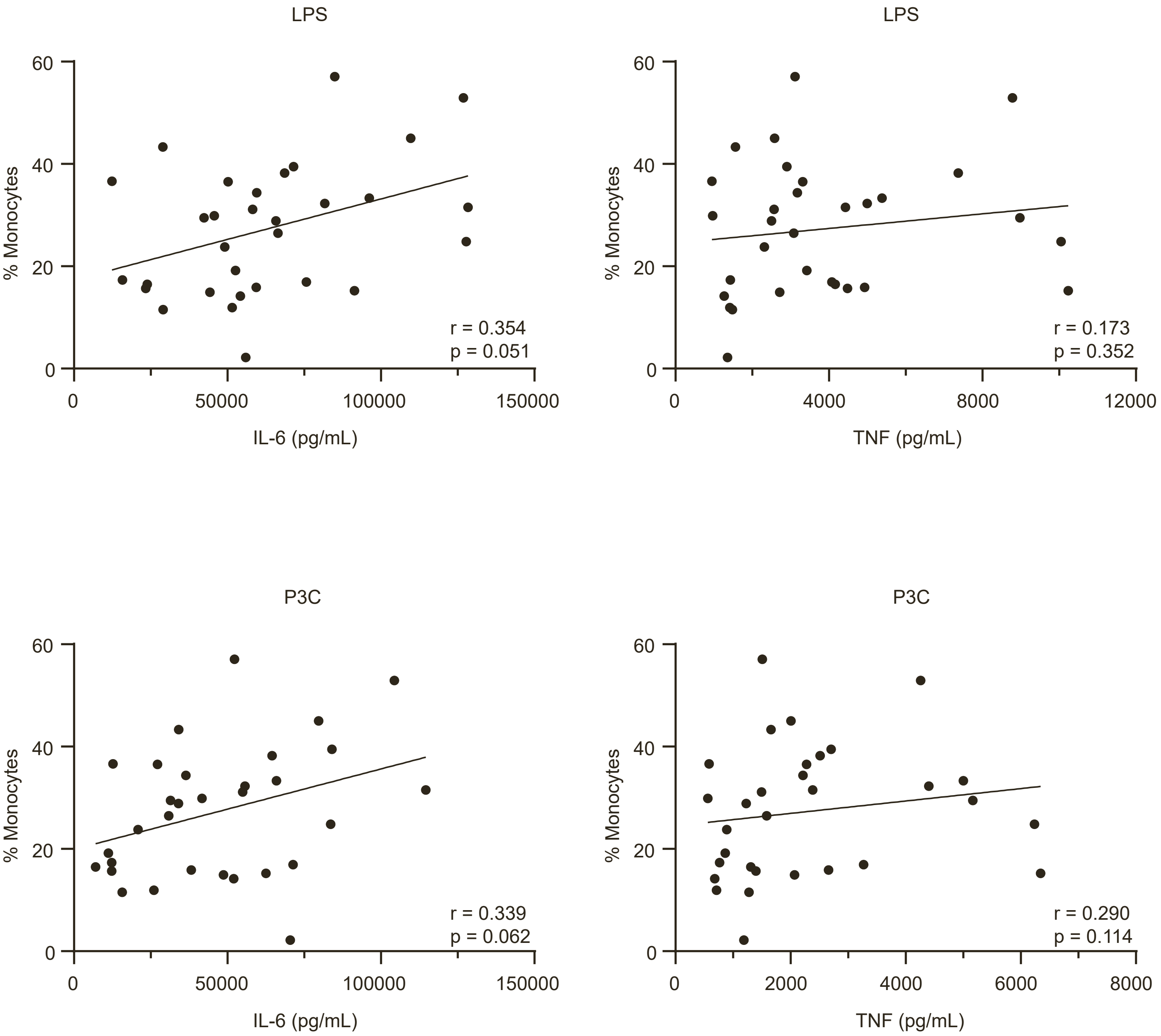

Figure S3: No significant correlation between the percentage of monocytes in PBMCs and IL-6 or TNF cytokine responses

Spearman's rho correlation between the percentage of monocytes in peripheral blood mononuclear cells (PBMCs) and interleukin 6 (IL-6) and tumor necrosis factor (TNF) production by PBMCs from hemodialysis (HD) patients (n=26) that were incubated for 24 hours with lipopolysaccharide (LPS) or Pam3CSK4 (P3C).

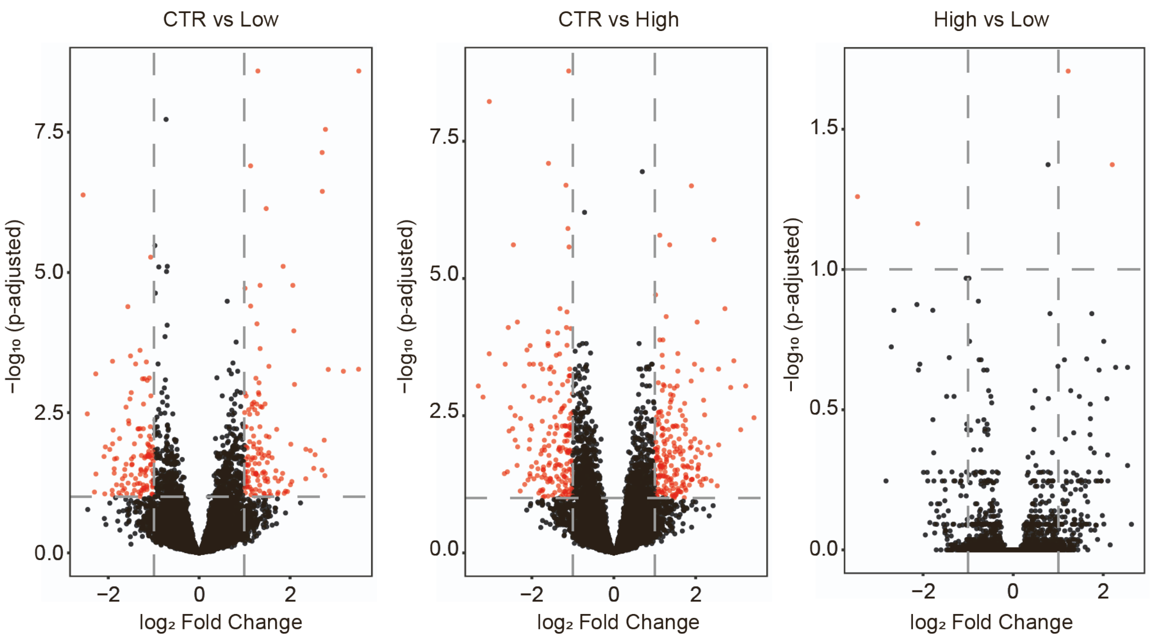

Figure S4: Effects of hemodialysis on transcriptional profiles of monocytes compared to healthy controls.
Volcano plot indicating RNA expression in monocytes from healthy controls versus patients with a low tumor necrosis factor (TNF) and interleukin 6 (IL-6) cell response to lipopolysaccharide (LPS) or Pam3CSK4 (P3C), healthy controls versus patients with a high TNF and IL-6 cell response to LPS or Pam3CSK4, and patients with a high TNF and IL-6 cell response to LPS or Pam3CSK4. Fold change (FC) and p-adjusted were calculated using analysis with DESeq2 (n=5 per group). Dotted lines indicate a FC of > 2 or < -2, and p-adjusted < 0.1.

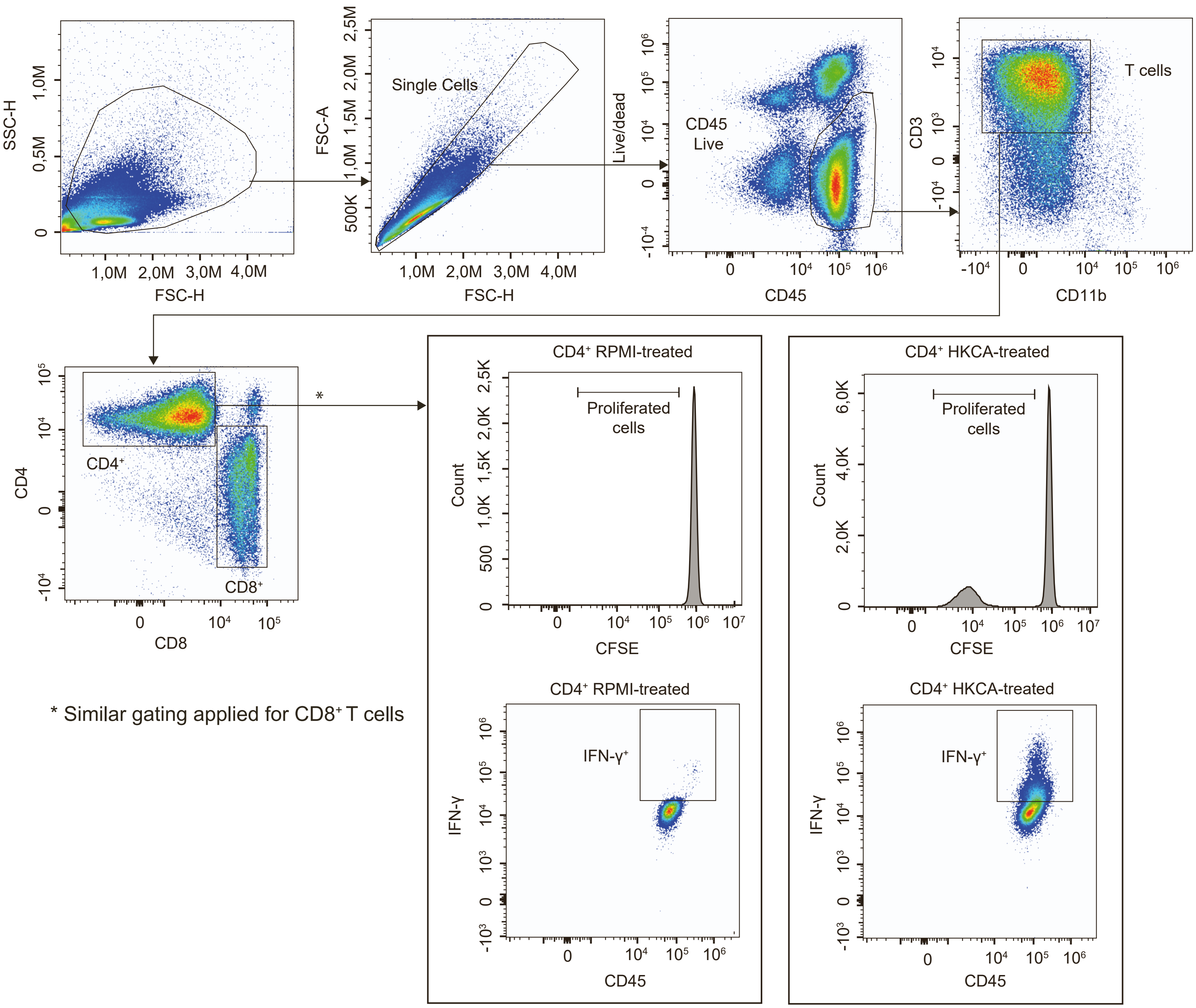

Figure S5: Gating strategy for determining proliferation and IFN-γ production in T cells using flow cytometry

A. Gating strategy applied to determine proliferation and production of interferon gamma (IFN-γ) by T cells present in monocyte:T cell co-cultures 6 days after stimulation with heat-killed Candida albicans (HKCA), Bacille Calmette Guérin (BCG) vaccine or RPMI (control), using flow cytometry. Example shows proliferation and IFN-γ production in CD4+ T cells of co-cultures stimulated with RPMI versus HKCA. The same gating strategy was applied to determine proliferation and IFN-γ in CD8+ T cells.

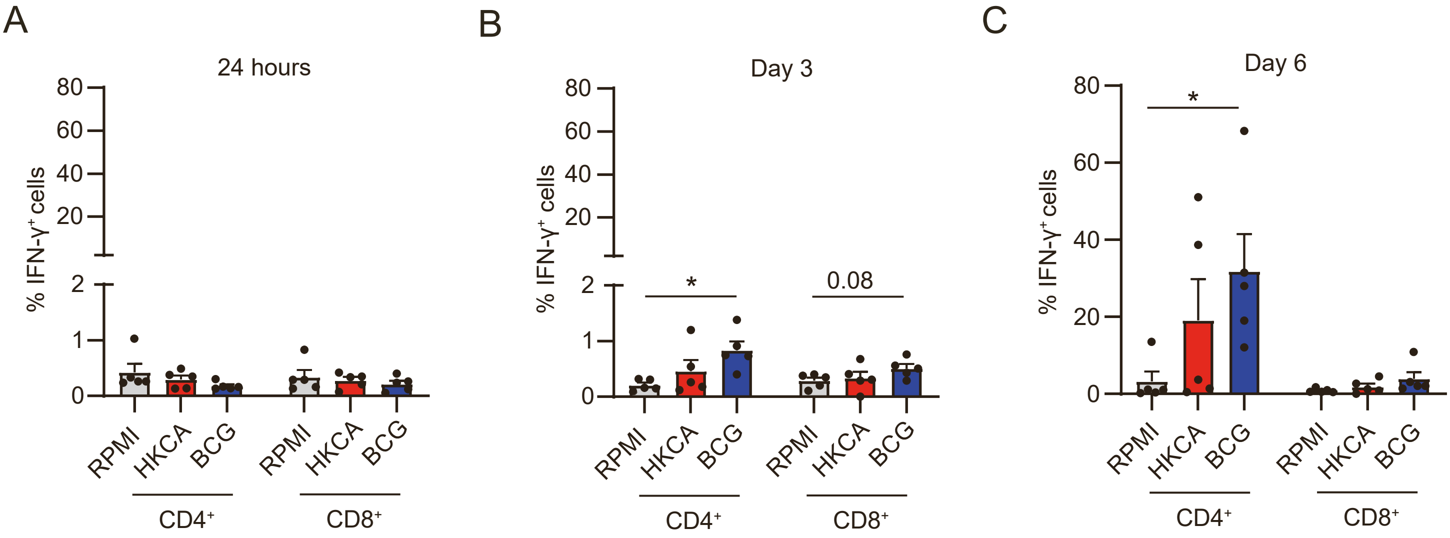

Figure S6: Production of IFN-γ following stimulation with HKCA, BCG and RPMI

A. Interferon gamma (IFN-γ) production by CD4+ and CD8+ T cells in PBMCs stimulated for 24h with heat-killed Candida albicans (HKCA), Bacille Calmette Guérin (BCG) vaccine or RPMI (control), measured 24 hours after stimulation (n=5 donors).

B. IFN-γ production by CD4+ and CD8+ T cells in PBMCs stimulated for 24h with heat-killed Candida albicans (HKCA), Bacille Calmette Guérin (BCG) vaccine or RPMI (control), measured 3 days after stimulation (n=5 donors).

C. IFN-γ production by CD4+ and CD8+ T cells in PBMCs stimulated for 24h with heat-killed Candida albicans (HKCA), Bacille Calmette Guérin (BCG) vaccine or RPMI (control), measured 6 days after stimulation (n=5 donors).

Mean ± SEM. * $p < 0.05$. Paired One-Way ANOVA with Dunnett's post-test (A-C).

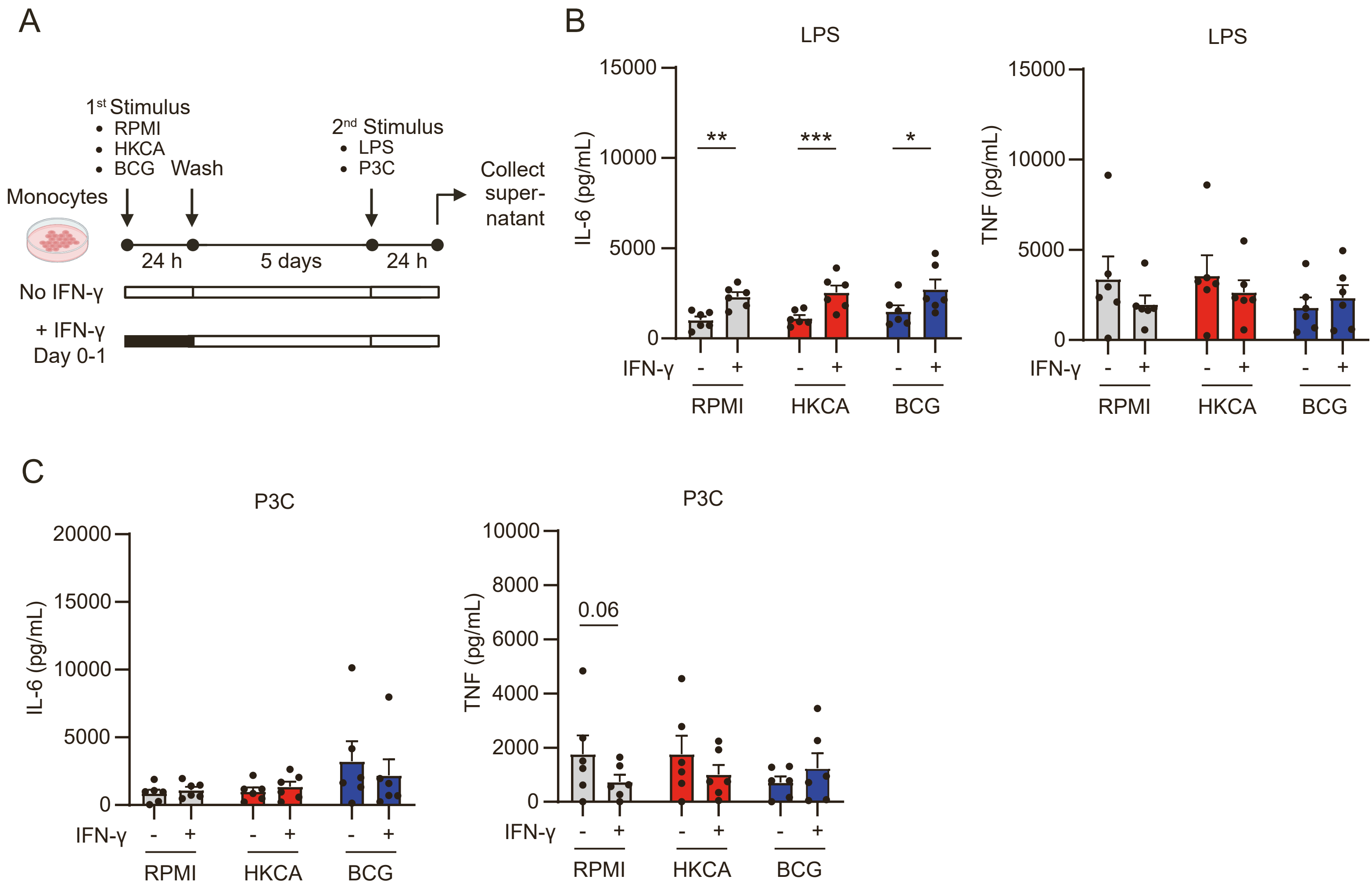

Figure S7: Short-term IFN-γ stimulation enhances LPS-induced IL-6 production in monocytes.
A. Schematic representation of in vitro trained immunity assays in which purified monocytes were trained with heat-killed Candida albicans (HKCA), Bacille Calmette Guérin (BCG) vaccine or RPMI (control) in presence or absence of interferon gamma (IFN-γ), and restimulated with RPMI, lipopolysaccharide (LPS) or Pam3CSK4 (P3C).
B, C. Interleukin 6 (IL-6) and Tumor Necrosis Factor (TNF) production in purified monocytes stimulated with RPMI, HKCA, or BCG vaccine in the presence or absence of IFN-γ for 24h, and restimulated with LPS (B) or Pam3CSK4 (C) 6 days later (n=6 donors).
Mean ± SEM. * $p < 0.05$, ** $p < 0.01$, *** $p < 0.001$; Paired t-tests on log10-transfromed cytokine concentrations.
